# Erratum

**Published:** 1827-01

**Authors:** 


ERRATUM.—In page 4 of the Advertisements, No. IX.
*for* "Mr. Grainger, 30, Broad-street, Bloomsbury,"
*read* "Mr. Grainger, 30, Broad-street-Buildings."

